# Supplementary material for: End Sequence Analysis Toolkit (ESAT) expands the extractable information from single-cell RNA-seq data
Source: Genome Res. 2016 Oct;26(10):1397–410. doi: 10.1101/gr.207902.116 (PMC5052061; doi:10.1101/gr.207902.116)
Supplement: Supplemental Material [file supp_gr.207902.116_Supp_Materials.tar.gz › ESAT-esat_v0.1/doc/html/classbroad_1_1pda_1_1seq_1_1protection_1_1_two_sample_scan_skellam_peak_caller-members.html]

ESAT: Member List


ESAT


- Main Page
- Related Pages
- Packages
- Classes
- Files

- Class List
- Class Index
- Class Hierarchy
- Class Members

All Classes Namespaces Files Functions Variables Enumerator Pages

- broad
- pda
- seq
- protection
- TwoSampleScanSkellamPeakCaller

broad.pda.seq.protection.TwoSampleScanSkellamPeakCaller Member List

This is the complete list of members for broad.pda.seq.protection.TwoSampleScanSkellamPeakCaller, including all inherited members.

|  |  |  |
| --- | --- | --- |
| backgroundData | broad.pda.seq.protection.TwoSampleScanSkellamPeakCaller | protected |
| backgroundName | broad.pda.seq.protection.TwoSampleScanSkellamPeakCaller | protected |
| backgroundProcessor | broad.pda.seq.protection.TwoSampleScanSkellamPeakCaller | protected |
| backgroundScanPvalues | broad.pda.seq.protection.TwoSampleScanSkellamPeakCaller | protected |
| EXPRESSION\_PVALUE\_CUTOFF | broad.pda.seq.protection.TwoSampleScanSkellamPeakCaller | static |
| filterWindows(Collection< Annotation > windows) | broad.pda.seq.protection.TwoSampleScanSkellamPeakCaller |  |
| genes | broad.pda.seq.protection.TwoSampleScanSkellamPeakCaller | protected |
| getPoissonLambda(int regionLength, double d, int windowSize) | broad.pda.seq.protection.TwoSampleScanSkellamPeakCaller | static |
| getSkellamPvalue(double backgroundLambda, double signalLambda, int backgroundCount, int signalCount) | broad.pda.seq.protection.TwoSampleScanSkellamPeakCaller | protectedstatic |
| logger | broad.pda.seq.protection.TwoSampleScanSkellamPeakCaller | protectedstatic |
| MIN\_WINDOW\_COUNT | broad.pda.seq.protection.TwoSampleScanSkellamPeakCaller | protectedstatic |
| pValueCutoffScan | broad.pda.seq.protection.TwoSampleScanSkellamPeakCaller | protected |
| pValueCutoffSkellam | broad.pda.seq.protection.TwoSampleScanSkellamPeakCaller | protected |
| signalData | broad.pda.seq.protection.TwoSampleScanSkellamPeakCaller | protected |
| signalName | broad.pda.seq.protection.TwoSampleScanSkellamPeakCaller | protected |
| signalProcessor | broad.pda.seq.protection.TwoSampleScanSkellamPeakCaller | protected |
| stepSize | broad.pda.seq.protection.TwoSampleScanSkellamPeakCaller | protected |
| transcriptomeSpace | broad.pda.seq.protection.TwoSampleScanSkellamPeakCaller | protected |
| trimPeak(Gene window) | broad.pda.seq.protection.TwoSampleScanSkellamPeakCaller | protected |
| trimPeakByQuantile | broad.pda.seq.protection.TwoSampleScanSkellamPeakCaller | protected |
| windowSize | broad.pda.seq.protection.TwoSampleScanSkellamPeakCaller | protected |
| writeResult(Collection< Annotation > windows, FileWriter writer) | broad.pda.seq.protection.TwoSampleScanSkellamPeakCaller |  |
| writeResults(Collection< Annotation > windows, String out) | broad.pda.seq.protection.TwoSampleScanSkellamPeakCaller |  |


---

Generated on Fri Aug 15 2014 15:11:03 for ESAT by  

 1.8.7
